# Parvalbumin Neurons in Zona Incerta Regulate Itch in Mice

**DOI:** 10.3389/fnmol.2022.843754

**Published:** 2022-03-01

**Authors:** Jiaqi Li, Yang Bai, Yi Liang, Yiwen Zhang, Qiuying Zhao, Junye Ge, Dangchao Li, Yuanyuan Zhu, Guohong Cai, Huiren Tao, Shengxi Wu, Jing Huang

**Affiliations:** ^1^Department of Neurobiology, School of Basic Medicine, Fourth Military Medical University, Xi’an, China; ^2^Department of Neurosurgery, General Hospital of Northern Theater Command, Shenyang, China; ^3^The Cadet Team 6 of School of Basic Medicine, Fourth Military Medical University, Xi’an, China; ^4^Department of Spine Surgery, Shenzhen University General Hospital, Shenzhen, China

**Keywords:** zona incerta, parvalbumin, itch, fiber photometry, chemogenetics

## Abstract

Pain and itch are intricately entangled at both circuitry and behavioral levels. Emerging evidence indicates that parvalbumin (PV)-expressing neurons in zona incerta (ZI) are critical for promoting nocifensive behaviors. However, the role of these neurons in itch modulation remains elusive. Herein, by combining FOS immunostaining, fiber photometry, and chemogenetic manipulation, we reveal that ZI PV neurons act as an endogenous negative diencephalic modulator for itch processing. Morphological data showed that both histamine and chloroquine stimuli induced FOS expression in ZI PV neurons. The activation of these neurons was further supported by the increased calcium signal upon scratching behavior evoked by acute itch. Behavioral data further indicated that chemogenetic activation of these neurons reduced scratching behaviors related to histaminergic and non-histaminergic acute itch. Similar neural activity and modulatory role of ZI PV neurons were seen in mice with chronic itch induced by atopic dermatitis. Together, our study provides direct evidence for the role of ZI PV neurons in regulating itch, and identifies a potential target for the remedy of chronic itch.

## Introduction

Itch is an uncomfortable sensation that triggers the desire to scratch (Davidson and Giesler, 2010). While acute itch protects our body from irritants by scratching, chronic itch, often associated with dermatologic, neuropathic, psychogenic, and systemic disorders, leads to the vicious itch-scratch cycle. Chronic itch, affecting nearly 15% of the population, is the most frequent cause of visits to dermatologists and exerts a huge personal and economic burden (Greaves and Khalifa, 2004; Weisshaar and Matterne, 2016). Despite substantial antipruritics available in the clinic, optimal therapeutic effect is hampered by our inadequate understanding of itch pathophysiology (Tey and Yosipovitch, 2011).

It has long been appreciated that pain and itch are intricately entangled at both circuitry and behavioral levels, and prior efforts have been made to distinguish itch circuitry from that of pain at the level of primary afferents and spinal dorsal horn (SDH) (Dong and Dong, 2018; Koch et al., 2018; Chen, 2021). Accumulating imaging data have indicated that the interwoven network involved in itch processing, including primary sensory cortex (S1), limbic system, and thalamus, mimics the “pain matrix” (Treede et al., 1999; Drzezga et al., 2001; Schweinhardt and Bushnell, 2010; Papoiu et al., 2012). Emerging studies aiming at elucidating supraspinal itch circuitry have also identified the role of the ventral tegmental area (Yuan et al., 2018; Su et al., 2019), central amygdala (Sanders et al., 2019; Samineni et al., 2021), and periaqueductal gray (PAG) (Gao et al., 2019; Samineni et al., 2019) in itch modulation, all of which are involved in pain regulation (Peters et al., 2021; Tan and Kuner, 2021). Thus, brain mechanisms that signal itch and pain appear remarkably similar.

As an inhibitory subthalamic nucleus, zona incerta (ZI) integrates various sensory modalities and feeds them to a series of downstream areas, including the thalamus, hypothalamus, midbrain, and spinal cord, to modulate behavioral outputs and convey motivational states (Mitrofanis, 2005; Wang et al., 2020b). As yet, ZI has been demonstrated to regulate fear memory (Zhou et al., 2018; Venkataraman et al., 2019), defensive behavior (Chou et al., 2018; Wang et al., 2019), predatory hunting (Zhao et al., 2019), anxiety (Li et al., 2021), and sleep (Liu et al., 2017). Interestingly, ZI is also implicated in pain modulation. Neuropathic pain is related with decreased GABAergic neuronal activity in ZI, and pharmacological activation of ZI improves neuropathic pain behaviors in rats (Masri et al., 2009; Petronilho et al., 2012; Moon et al., 2016; Moon and Park, 2017). Considering that ZI contains heterogeneous groups of cell with diverse functions (Mitrofanis, 2005; Wang et al., 2020b). Wang et al. (2020a) further investigated the role of parvalbumin (PV)-expressing neurons, a neuronal subset mainly residing in the ventral ZI (ZIv), in pain modulation, and observed that these neurons promote nocifensive behaviors. Nevertheless, it still remains unknown how ZI PV neurons encode and modify itch processing. Given the similarity in brain circuitry for pain and itch, we propose this neuronal subpopulation may participate in itch modulation.

Herein, we used morphological and optical imaging approaches, as well as chemogenetic manipulations to identify neural dynamics and functional roles of ZI PV neurons during itch processing in mice. The results showed that activation of these neurons inhibited scratching behaviors in mouse models of acute and chronic itch. Our results highlight novel cellular mechanisms of a diencephalic area underlying itch signaling and brain modulation of itch-related scratching behaviors.

## Materials and Methods

### Experimental Animals

All mice used in this study were adult male with a pure C57BL/6J background. C57BL/6J mice were provided by Experimental Animal Center of the Fourth Military Medical University. PV-IRES-Cre (Stock No: 008069) mice were acquired from the Jackson Laboratory and crossed with wild-type C57BL/6J mice. Mice aged 9–10 weeks were used for FOS immunostaining, while those aged 7–8 weeks received virus injection for photometry recordings and chemogenetic manipulations. All tests were performed during the light phase. The experimenters were blinded to the genotype and experimental conditions. All mice were housed under a 12-h light/dark cycle at 22–25°C with *ad libitum* access to food and water under environmentally controlled conditions. All procedures were approved by the Institutional Animal Care and Use Committee of the Fourth Military Medical University and conformed to the Guide for the Care and Use of Laboratory Animals published by the National Institutes of Health.

### Itching Models

For pruritogen-induced acute itch, the mice were shaved on the back of neck and received intradermal injection of histamine (10 μg/μL, Cat#: H7125, Sigma), chloroquine (10 μg/μL, Cat#: C6628, Sigma), or vehicle (saline) into the nape of neck with a total volume of 10 μL. The scratching behaviors were recorded with a digital camera for 30 min and the scratching bouts were counted in a blind manner.

Tropical application of calcipotriol to mouse skin recapitulated features of atopic dermatitis (AD) and was adopted in the present study as the chronic itch model (Kim et al., 2019). As previously reported (Kim et al., 2013), bilateral ear skin were tropically treated with the calcipotriol scalp solution (1.5 mg/30 mL, 30 μL per side, LEO Laboratories Limited, Madison, NJ, United States) or vehicle (ethanol) for three consecutive weeks. Scratching behaviors were video recorded for 30 min after the final drug application to verify successful establishment of the model.

### Stereotaxic Surgery

Mice were anesthetized with isoflurane (4% for induction and 1.5% for maintenance) and then mounted in a stereotaxic frame (RWD Life Science Inc., Shenzhen, China). The skull was exposed with a small incision and holes were drilled. Injection into ZIv was performed using a microinjection needle with a 10 μL microsyringe (Shanghai Gaoge Industry and Trade Co., Ltd., Shanghai, China) to deliver the virus at a rate of 30 nL/min using a microsyringe pump (Kd Scientific Inc., Holliston, MA., United States). The microsyringe had a glass pipette of 15–25 μm in diameter at the tip to avoid excessive tissue injury. Following injection, the needle was left in place for another 10 min before retraction. According to the Paxinos and Franklin mouse brain atlas (4th edition), the stereotaxic coordinates for virus injection in ZIv were set as follows: anterior posterior (AP), −2.46; medial lateral (ML), 1.50; and dorsal ventral (DV), −4.25 mm. For fiber photometry, fiber implantation (230 μm OD, 0.5 NA, Newdoon) was performed immediately after viral injection, and the dental acrylic and skull-penetrating screws were used to support the ceramic ferrule. The stereotaxic coordinates for implantation in ZIv were as follows: AP, −2.46; ML, 1.50; and DV, −4.23 mm.

For chemogenetic manipulation of ZI PV neurons, a 200 nL mixture of rAAV-EF1α-DIO-hM3D(Gq)-mCherry-WPREs (titer: 2.36 × 10^12^ vg/mL; Cat#: PT-0042, BrainVTA, China) or rAAV-EF1α-DIO-hM4D(Gi)-mCherry-WPREs (titer: 2.40 × 10^12^ vg/mL; Cat#: PT-0043, BrainVTA) was injected bilaterally into the ZI in PV-Cre mice, with rAAV-hSyn-DIO-EGFP-WPRE-hGHpA injected as the control. For recording the activity of ZI PV neurons, 200-nL of rAAV-hSyn-DIO-GCaMP6s-WPREs-pA (titer: 5.40 × 10^12^ vg/mL; Cat#: PT-0091, BrainVTA) or rAAV-hSyn-DIO-EGFP-WPRE-hGHpA (titer: 2.31 × 10^12^ vg/mL; Cat#: PT-1103, BrainVTA) was injected into the right ZI of PV-Cre mice.

### Chemogenetic Manipulations and Behavioral Tests

The mice were allowed to recover for 3 weeks after chemogenetic virus injection before behavioral experiments. The mice were handled and acclimatized to the testing apparatus for at least 3 days prior to performing behavior tests. For acute itch stimuli, the mice were firstly intraperitoneally injected with 80 μL clozapine N-oxide (CNO, 0.75 mg/mL, dissolved in saline, Cat#: 4936, Tocris, Bristo, United Kingdom). 40 min later, the mice received intradermal injection of histamine or chloroquine, and then were immediately transferred back to the recording cages. Then, the animals were videotaped for at least 30 min. The number of scratching behavior was manually counted. After behavioral tests, the mice were immediately perfused and those with viral expression restricted in the ZI were chosen for statistical analysis. FOS immunostaining was then performed to validate the efficacy of chemogenetics.

For chronic itch stimuli, the establishment of AD model was initiated immediately after viral injection. On the day of behavioral experiments, the mice were treated with CNO, and then placed in the recording cages where the recording started 40 min later and continued for 30 min. Other procedures were the same as those described under the condition of acute itch.

### Fiber Photometry

The mice were allowed to recover for 3 weeks after the stereotaxic surgery. The mice were handled and acclimatized to the testing apparatus for at least 3 days prior to experimentation. For acute itch stimuli, the mice received histamine or chloroquine injection and then were immediately transferred back to the recording apparatus on the day of experimentation. For chronic itch stimuli, the establishment of AD model was performed immediately after viral injection. On the day of behavioral test, the mice were placed in the recording apparatus. Fluorescence signals produced by a 473 nm laser (OBIS 488LS; Coherent) was reflected by a dichroic mirror (MD498; Thorlabs, Inc., Newton, MA, United States), focused by a 0.3 NA x10 objective lens (Olympus, Japan), and coupled to an optical commutator (Doris Lenses, Canada). An optical fiber (230 μm OD, 0.5 NA) guided the light between the implanted optical fiber and the commutator. The laser power was adjusted to 0.01–0.02 mW at the tip of the optical fiber for minimizing bleaching of the GCaMP6s probes. The GCaMP6s fluorescence signals were bandpass filtered (MF525-39, Thorlabs, Inc., Newton, MA, United States), and an amplifier was used to convert the CMOS (DCC3240M, Thorlabs, Inc., Newton, MA, United States) current output to a voltage signal. The voltage signal was further filtered through a low-pass filter (40 Hz cutoff, ThinkerTech). The analog voltage signals were digitalized at 50 Hz and recorded by the multichannel fiber photometry recording system (ThinkerTech). The fluorescence signals were recorded continuously during scratching. After behavioral tests, the mice were perfused. Viral injection and fiber implantation were confirmed *post doc*, and animals with incorrect locations were excluded from final analyses.

For data analysis, fluorescence change (ΔF/F) was represented by (F-F_0_)/F_0_. F_0_ referred to the median of the fluorescence values in the baseline period, while F referred to the fluorescence values of each time point. The ΔF/F values of mice in each group were then averaged. To precisely analyze the change in fluorescence values across the scratching train, the baseline period and the post-scratching period were defined as −2 to −1 s relative to the scratching onset and 0–3 s after the onset of scratching behaviors, respectively. The areas under the curve (AUC) of ΔF/F in each time window defined were also adopted to quantify the change of fluorescence values induced by scratching. In addition, permutation tests were performed for analyzing the statistical significance of the event-related fluorescence change (ERF) (Li et al., 2016). Permutation tests with 1000 permutations were used to compare the fluorescence change at each time point of events with the baseline ERF. A series of statistical *P*-values at each time point were then generated and the statistical results were superimposed on the average ERF curve with red segments indicating statistically significant (*P* < 0.05) increase. Non-significant changes were shown as black lines.

### Immunofluorescent Staining

The mice were anesthetized with overdose of 2% pentobarbital sodium, and perfused transcardially with 20 ml of 0.01 M phosphate buffer saline (PBS, pH 7.4), followed by 100 ml 4% paraformaldehyde (PFA) fixative solution in 0.1 M phosphate buffer (PB, pH 7.4). After perfusion, their brains were removed, placed in 30% sucrose solution for 24 h at 4°C, and then cut into coronal sections at 30 μm thickness with a cryostat (Leica CM 1950, Leica Microsystems Inc., United States). The sections containing ZI were used for immunostaining. Briefly, the sections were incubated in 10% normal donkey serum (NDS) for 40 min at room temperature (RT) to block non-specific immunoreactivity, and then incubated with primary antibodies at 4°C overnight and secondary antibodies at RT for 4 h in sequence. Finally, the sections were air-dried and cover-slipped with a mixture of 0.1% (v/v) DAPI (Cat#: d9564, Sigma), 50% (v/v) glycerol and 2.5% (w/v) triethylenediamine in 0.01 M PBS. Photomicrographs for injection sites were taken using Olympus VS200 microscope (10×), and confocal images were taken using Olympus FV3000 microscope. Cell counting was carried out manually in a blinded manner.

For examination of FOS expression in the ZI, C57BL/6J mice received intradermal injection of histamine or chloroquine, and then were placed into original rearing cages for 90 min before being perfused. The AD model mice were acclimatized in the rearing cages for 90 min and then sacrificed. The primary antibodies used for double immunofluorescence staining of FOS and PV were mouse anti-PV (1:200, Cat#: p3088, Sigma) and rabbit anti-c-fos (1:500, Cat#: 226008, Synaptic systems). The secondary antibodies were donkey anti-mouse IgG-Alexa 594 (1:500, Cat#: A21203, Invitrogen) and donkey anti-rabbit IgG-Alexa 488 (1:500, Cat#: ab150073, Abcam). For the analysis of FOS expression in different ZI subregions, 10 × confocal images of the ZI area were taken, and the number of FOS-immunoreactive (ir) neurons in the rostral ZI (ZIr), ZIv, the dorsal ZI (ZId), and the caudal ZI (ZIc) was counted from each mouse (three sections for each region per mouse) in different groups. For the analysis of FOS expression in ZI PV neurons, 20 × confocal images were taken, and the number of neurons expressing FOS or PV were counted from three sections for each mouse.

For verifying the specificity of GCaMP6s expression in PV neurons, PV immunostaining was performed in the ZI from three sections randomly selected from each mouse (*n* = 3) transduced with the Cre-dependent GCaMP6s protein. The primary antibody and the second antibody were rabbit anti-PV (1:200, Cat#: ab11427, Abcam) and donkey anti-rabbit IgG-Alexa 594 (1:500, Cat#: A21206, Invitrogen), respectively. 20 × confocal images were taken and the number of neurons expressing GCaMP6s-mCherry or PV was counted.

To determine whether hM3Dq was selectively expressed in PV neurons, PV immunostaining was performed in the ZI from three sections randomly selected from each mouse (*n* = 3) transduced with the Cre-dependent hM3Dq protein. The primary antibody and the second antibody were rabbit anti-PV and donkey anti-rabbit IgG-Alexa 488, respectively. 20 × confocal images were taken and the number of neurons expressing hM3Dq-mCherry or PV was counted.

For verifying the efficacy of chemogenetics, FOS immunostaining was performed in the ZI from three sections randomly selected from each mouse (*n* = 3) transduced with the Cre-dependent hM3Dq-mCherry or mCherry alone. The primary antibodies used was rabbit anti-c-fos, and the secondary antibody was donkey anti-rabbit IgG-Alexa 488. 20× confocal images were taken and the number of neurons expressing hM3Dq-mCherry or FOS was counted.

### Statistical Analysis

All data were expressed as the mean ± SEM. Statistical tests were performed using GraphPad Prism 7 and MATLAB 2018b (MathWorks). The normality test were performed using Kolmogorov–Smirnov test. For the analysis of immunofluorescent and behavioral data, one-way ANOVA with a *post hoc* least significant difference (LSD) test, Kruskal–Wallis test and Nemenyi multiple comparisons tests, or an unpaired *t*-test was used. For fiber photometry data, repeated measure ANOVA was firstly performed. If significant main effects were found, a simple effect analysis was performed. *P* < 0.05 was considered statistically significant.

## Results

### Increased FOS Expression in the Ventral ZI Neurons Under the Condition of Acute and Chronic Itch Stimuli

**FIGURE 1:**
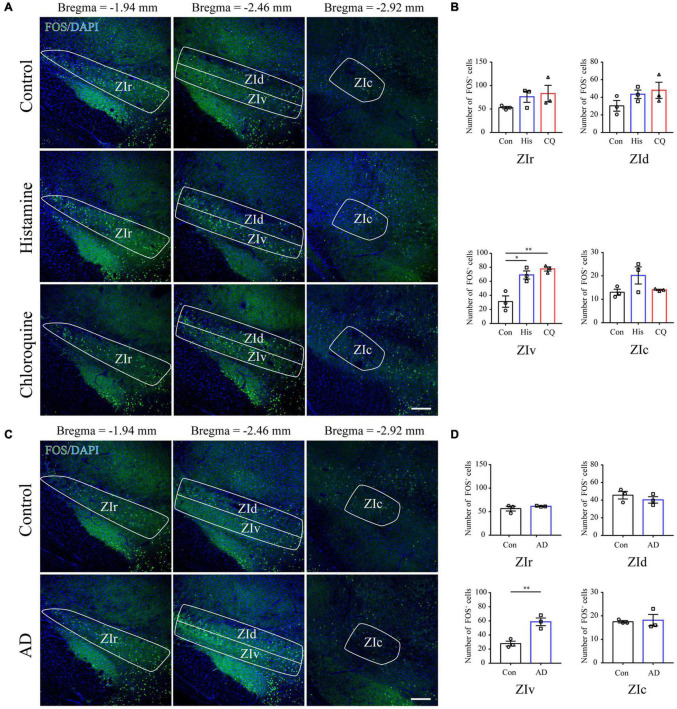
Acute itch stimuli and Chronic itch stimuli increase FOS expression in the ZIv. **(A)** Representative images of FOS staining in the ZI after saline (the upper panel), histamine (the middle panel), and chloroquine (the lower panel) injection. Scale bar = 200 μm. **(B)** Quantification of FOS-expressing neurons in response to saline, histamine, and chloroquine injection showed both histamine and chloroquine stimuli increased FOS expression in the ZIv instead of other ZI subregions. *n* = 3 mice per group, three sections per mouse. One-way ANOVA with *post hoc* LSD test for multiple comparisons. For the analysis in ZIv, *F*(2,6) = 17.378, *P* = 0.003. **(C)** Representative images of FOS staining in the ZI in control (the upper panel) and AD (the lower panel) mice. Scale bar = 200 μm. **(D)** Quantification of FOS-expressing neurons in control and AD mice showed AD increased FOS expression in the ZIv instead of other ZI subregions. *n* = 3 mice per group, three sections per mouse. Unpaired *T*-test. For the analysis in ZIv, *t* = –4.759, *P* = 0.009. ZIr, the rostral ZI; ZIv, the ventral ZI; ZId, the dorsal ZI; ZIc, caudal ZI; AD, atopic dermatitis. ^∗^*P* < 0.05, ^∗∗^*P* < 0.01.

FOS is widely utilized as a biomarker of neuronal activation upon external sensory stimuli (Coggeshall, 2005). Herein, we performed FOS immunostaining in brain sections containing the ZI 90 min after acute itch stimuli, or 21 days after the initiation of calcipotriol administration when the mice exhibited robust spontaneous scratching behavior. The successful establishment of acute (Supplementary Figures 1A,B) and chronic (Supplementary Figures 1C,D) models was verified by recordings of mouse scratching behavior. Since the ZI is a long diencephalic nucleus along the rostro-caudal axis, we counted the number of FOS-ir neurons in the rostral, dorsal, ventral, and caudal parts of ZI in mice among control, histamine and chloroquine groups. Quantification data showed that acute itch stimuli specifically increased the number of FOS-ir neurons in the ZIv (control: 31.17 ± 8.01, histamine: 69.00 ± 5.68, and chloroquine: 77.50 ± 2.93; *P* = 0.003) (Figures 1A,B), and no change in the number of FOS-expressing neurons was seen in other subregions. For chronic itch stimuli, a significant increase in FOS-expressing neurons was also seen in the ZIv (control: 27.83 ± 3.44, AD: 58.67 ± 5.49; *P* = 0.009), but not in other parts of ZI (Figures 1C,D). These results provide evidence for the activation of the ZIv upon both acute and chronic itch stimuli, laying morphological basis for subsequent investigations of ZI PV neurons in itch processing.

### Zona Incerta Parvalbumin Neurons Are Activated During Acute Itch Processing

**FIGURE 2:**
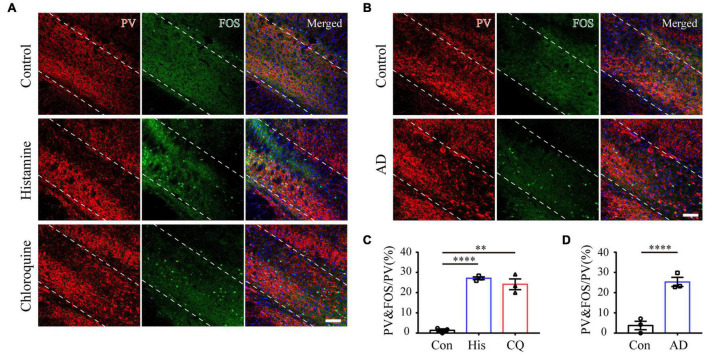
Acute and chronic itch stimuli increase FOS expression in ZI PV neurons. **(A–C)** Representative images of FOS and PV staining in ZI after saline (the upper panel), histamine (the middle panel), and chloroquine (the lower panel) injection. **(B)** Representative images of FOS and PV staining in ZI in control (the upper panel) and AD (the lower panel) mice. Scale bar = 100 μm. **(C)** Quantification of FOS-expressing neurons in response to saline, histamine, and chloroquine injections showed both histamine and chloroquine stimuli increased FOS expression in ZI PV neurons. *n* = 3 mice per group, three sections per mouse. One-way ANOVA with *post hoc* LSD test for multiple comparisons. *F*(2,6) = 73.642, *P* < 0.001. **(D)** Quantification of FOS-expressing neurons in control and AD mice showed chronic itch stimuli increased FOS expression in ZI PV neurons. *n* = 3 mice per group, three sections per mouse. Unpaired *T*-test. *t* = –7.03, *P* = 0.002. ^∗∗∗∗^*P* < 0.0001, ^∗∗^*P* < 0.01.

The ZI has a wealthy cluster of neuro-chemically distinct neurons, among which GABAergic PV neurons are mainly located in the ZIv (Mitrofanis, 2005). The modulatory role of ZI PV neurons in nocifensive behavior has been identified (Wang et al., 2020a). For these reasons, we focused on the role of this neuronal subpopulation in itch processing. A combination of PV and FOS immunostaining was firstly performed to test the activation of ZI PV neurons after introduction of acute itch stimuli. As illustrated in Figures 2A,C, the majority of these neurons were accumulated in the ZIv, with a few scattered in the ZId. Notably, a higher proportion of ZI PV neurons expressed FOS under the condition of both histamine and chloroquine stimuli, compared with the control group (control: 1.40 ± 0.72%, histamine: 27.12 ± 7.02%, and chloroquine: 24.14 ± 2.66%; *P* < 0.001).

**FIGURE 3:**
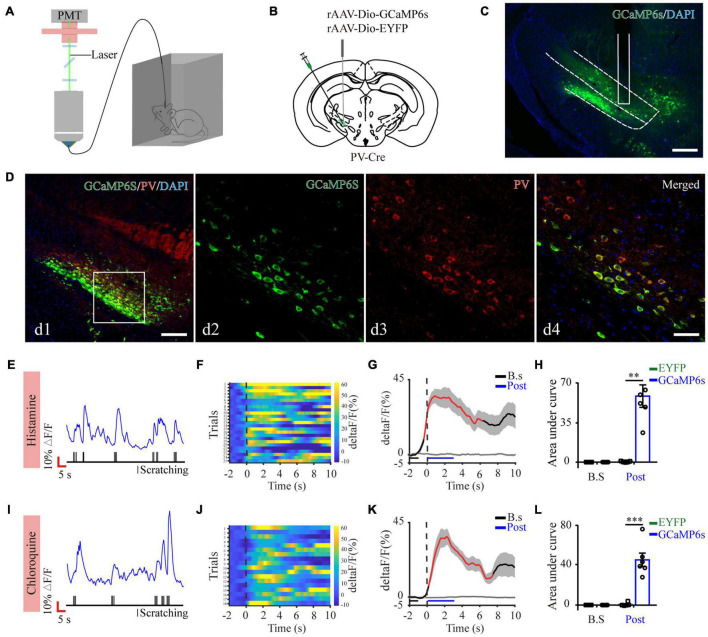
Increased activity of ZI PV neurons during acute itch-induced scratching behavior. **(A)** The fiber photometry setup. **(B)** Schematic showing the viral targeting of AAV-DIO-GCaMP6s-EYFP and AAV-DIO-EYFP into the ZI of PV-Cre mice. **(C)** Histological verification of viral expression (green) and optical fiber implantation in the ZI in a representative mouse. **(D)** Representative photographs showing the expression of PV (red) in GCaMP6s-positive (green) neurons in the ZI of PV-Cre mice. The framed area in d1 was magnified in d2-4. Scale bars represent 100 μm in d1 and 50 μm in d2-4. **(E)** Representative GCaMP6s fluorescence trace (top) and behavioral trace (bottom) recorded simultaneously in ZI PV neurons in response to histamine injection. **(F)** Heatmap illustrating GCaMP6s fluorescence aligned to the beginning of individual scratching trains in all the mice in response to histamine stimuli. Each row represents Ca^2+^ signals corresponding to one scratching train. The color scale at the right indicates ΔF/F. **(G)** Mean fluorescent signal in response to histamine stimuli in all the mice recorded, with shaded areas indicating the SEM. The black and gray lines represent the signals of PV ZI neurons in mice with AAV-GCaMP6s and AAV-EYFP injection, respectively. The red line represents statistically significant increase from the baseline (*P* < 0.05; multivariate permutation test). The vertical dotted line indicates the scratching bout. **(H)** Area under the curve showing fluorescence changes of ZI PV neurons in the mice with AAV-GCaMP6s and AAV-EYFP injection in both pre-scratching and post-scratching periods under the condition of histamine stimuli. Repeated ANOVA followed by simple effects analysis. *n* = 5 mice in EYFP group and 6 mice in GCaMP6s group, *F*(1,9) = 27.892, *P* = 0.001. **(I–L)** Same conventions as **(E–H)** but for recording in response to chloroquine stimuli. *n* = 5 mice in EYFP group and 6 mice in GCaMP6s group, *F*(1,9) = 36.586, *P* < 0.001. ^∗∗∗^*P* < 0.001, ^∗∗^*P* < 0.01. PMT, photomultiplier.

Next, to quantify physiological activities of these neurons related with acute itch-evoked scratching, we stereotaxically injected rAAVs expressing the Cre-dependent GCaMP6s (AAV-DIO-GCaMP6s) into the ZI of PV-Cre mice, with rAAVs containing only a fluorescent tag served as controls, and implanted an optical fiber with its tip situated in the ZI for long-term recordings of GCaMP6s fluorescence *via* fiber photometry (Figures 3A–C). GCaMP6s expression in PV neurons was confirmed *post doc* using PV immunostaining (Figure 3D), and 90.66 ± 2.38% GCaMP6s-expressing neurons were labeled by PV. Three weeks after virus injection, the mice were injected with histamine. By aligning GCaMP6s signals in time with scratching trains, we observed that the calcium fluorescence of ZI PV neurons of GCaMP6s group was significantly increased, which initiated at 0.10 ± 2.45 s and continued until 5.13 ± 1.57 s in the post-onset period, compared with that in control group (Figures 3E–H). To extend above observations to histamine-independent itch, we investigated the effect of chloroquine injection and obtained similar responses in these neurons. This increase occurred from 0.16 ± 0.28 s to 4.08 ± 0.71 s in the post-scratching period (Figures 3I–L). In summary, these data suggest that ZI PV neurons exhibit rapid and strong activation during acute itch-induced scratching.

### Chemogenetic Activation of Zona Incerta Parvalbumin Neurons Attenuates Acute Itch-Induced Scratching

**FIGURE 4:**
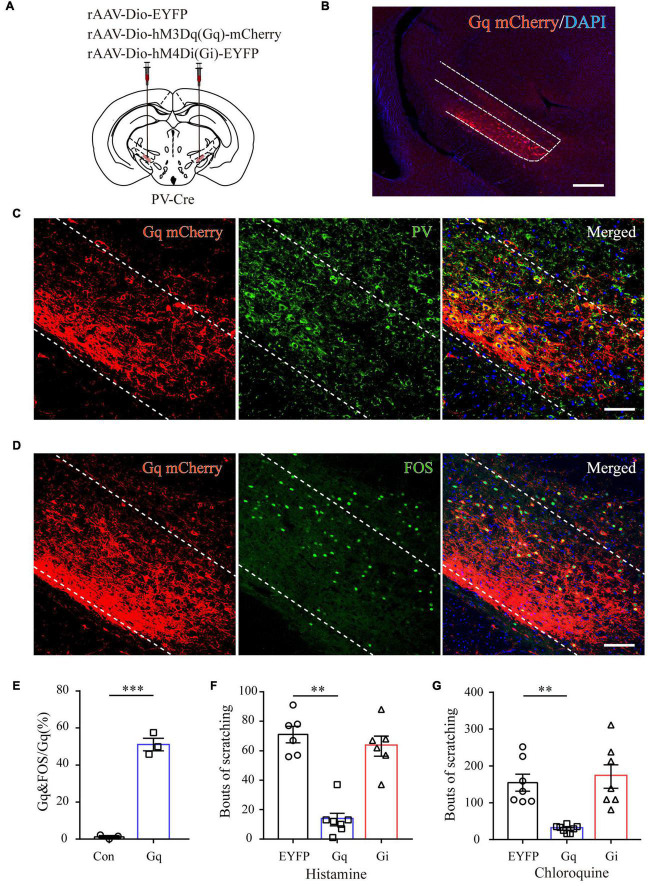
ZI PV neurons negatively regulate scratching behaviors during acute itch. **(A)** Schematic illustration of viral injection for chemogenetic modulation of ZI PV neurons. **(B)** Histological verification of viral expression in the ZI in a representative mouse with AAV-DIO-hM3Dq-mCherry injection. Scale bar = 250 μm. **(C)** Representative photographs showing the expression of PV (green) in hM3Dq-positive (red) neurons in the ZI of PV-Cre mice. **(D)** Representative photographs showing the expression of FOS (green) in hM3Dq-positive (red) neurons in the ZI of PV-Cre mice. **(E)** Quantification of FOS-expressing neurons in mice with AAV-hM3Dq and AAV-mCherry injection showed CNO injection increased FOS expression in ZI PV neurons in the mice in AAV-hM3Dq group. *n* = 3 mice per group, three sections per mouse. Unpaired *T*-test. *t* = –14.44, *P* < 0.001. **(F,G)** Chemogenetic activation of ZI PV neurons reduces scratching behaviors induced by histamine **(F)** and chloroquine **(G)**, while inhibition of them exerts no effect on scratching behaviors. One-way ANOVA with *post hoc* LSD test for multiple comparisons. For the analysis of histamine-induced itch, *F*(2,6) = 10.599, *P* = 0.001. *n* = 6 mice in EGFP and hM4Di groups and 7 in hM3Dq group. For the analysis of chloroquine-induced itch, *F*(2,6) = 13.341, *P* < 0.001. *n* = 6 mice in EGFP and hM4Di groups and 7 in hM3Dq group. *n* = 7 mice in EGFP and hM4Di groups and 8 in hM3Dq group. ^∗∗∗^*P* < 0.001, ^∗∗^*P* < 0.01.

To further characterize the precise role of ZI PV neurons in acute itch processing, chemogenetic manipulations of these neurons were performed. We targeted them by local injection of rAAVs delivering a construct containing excitatory (hM3Dq) or inhibitory (hM4Di) designer receptor fused with EYFP into bilateral ZI of PV-Cre mice. The mice injected with rAAV-DIO-EYFP served as controls (Figures 4A,B). Immunostaining results showed that 91.23 ± 3.78% mCherry-labeled neurons expressed PV in the ZI (Figure 4C). In addition, CNO treatment increased FOS expression in ZI PV neurons of hM3Dq group compared to control group (control: 1.25 ± 0.31%, hM3Dq: 51.14 ± 1.60%; *P* < 0.001) (Figures 4D,E). These immunostaining data suggest the reliability and efficiency of modulating the activity of ZI PV neurons with chemogenetics. Behavioral data showed that pharmacogenetic activation of ZI PV neurons greatly attenuated mouse scratching behavior induced by histamine (EYFP group: 71.00 ± 5.54, hM3Dq group: 13.28 ± 4.26, and hM4Di group: 63.17 ± 6.78; *P* = 0.001) and chloroquine (EYFP group: 154.71 ± 23.13, hM3Dq group: 29.00 ± 3.73, and hM4Di group: 171.43 ± 31.82; *P* < 0.001), whereas the inhibition of these neurons exerted no effect on scratching behavior (Figures 4F,G). Given above, ZI PV neurons negatively regulate scratching behaviors evoked by acute itch.

### Zona Incerta Parvalbumin Neurons Regulate Chronic Itch-Induced Scratching

**FIGURE 5:**
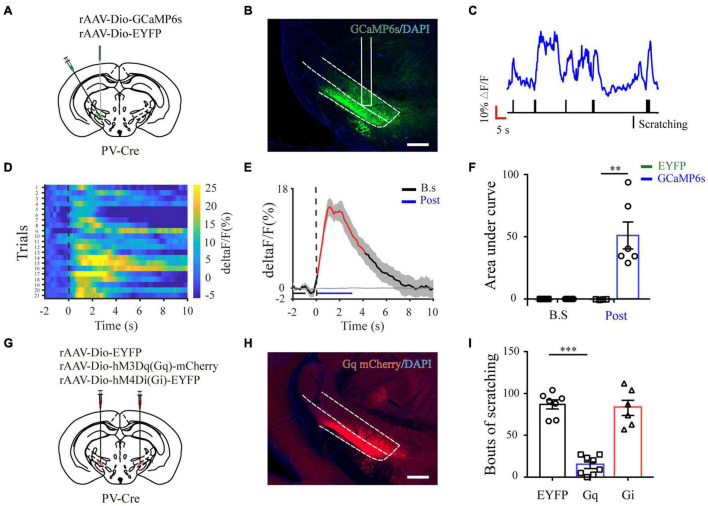
ZI PV neurons negatively regulate scratching behaviors during chronic itch. **(A)** Schematic showing the viral targeting of AAV-DIO-GCaMP6s-EYFP and AAV-DIO-EYFP into the ZI of PV-Cre mice with AD. **(B)** Histological verification of viral expression (green) and optical fiber implantation in the ZI in a representative mouse. **(C)** Representative GCaMP6s fluorescence trace (top) and behavioral trace (bottom) recorded simultaneously in ZI PV neurons in response to calcipotriol injection. **(D)** Heatmap illustrating GCaMP6s fluorescence aligned to the beginning of individual scratching trains in all the mice in response to calcipotriol stimuli. Each row represents Ca^2+^ signals corresponding to one scratching train. **(E)** Mean fluorescent signal in response to calcipotriol stimuli in all the mice recorded, with shaded areas indicating the SEM. The black and gray line represent the signals of PV ZI neurons in mice with AAV-GCaMP6s and AAV-EYFP injection, respectively. The red line represents statistically significant increase from the baseline (*P* < 0.05; multivariate permutation test). **(F)** Area under the curve showing fluorescence changes of ZI PV neurons in mice with AAV-GCaMP6s and AAV-EYFP injection in both pre-scratching and post-scratching periods under the condition of calcipotriol stimuli. Repeated ANOVA followed by simple effects analysis. *n* = 4 mice in EYFP group and 6 mice in GCaMP6s group, *F*(1,8) = 14.355, *P* < 0.01. **(G)** Schematic illustration of viral injection in mice with AD for chemogenetic modulation of ZI PV neurons. **(H)** Histological verification of viral expression within ZI in a representative mouse with AAV-DIO-hM3Dq-mCherry injection. Scale bar = 250 μm. **(I)** Chemogenetic activation of ZI PV neurons reduces scratching behaviors induced by calcipotriol injection, while the inhibition of them exerts no effect on scratching behaviors. One-way ANOVA with *post hoc* LSD test for multiple comparisons. *F*(2,6) = 49.27, *P* < 0.001. *n* = 7, 8, and 6 in EGFP, hM3Dq, and hM4Di group, respectively. ^∗∗∗^*P* < 0.001, ^∗∗^*P* < 0.01.

To further analyze whether ZI PV neurons are also involved in chronic itch, we established a mouse model of AD induced by calcipotriol. Immunostaining results showed that chronic itch stimuli increased FOS expression in ZI PV neurons (control: 3.77 ± 2.07%, AD: 25.29 ± 2.25%; *P* = 0.002) (Figures 2B,D). We obtained similar results as in acute itch using fiber photometry, indicating that ZI PV neurons displayed a rapid increase in GCaMP6s fluorescence which closely matched with the onset of each scratching train (from 0.78 ± 0.18 s to 7.36 ± 1.42 s in the post-scratching period) in mice with AD (Figures 5A–F and Supplementary Figure 2A). As expected, chemogenetic activation of ZI PV neurons induced an apparent decrease in the number of scratching in AD mice, while the inhibition of these neurons did not influence the scratching behavior (EYFP group: 87.00 ± 5.37, hM3Dq group: 14.25 ± 3.95, and hM4Di group: 82.83 ± 8.36; *P* < 0.001) (Figures 5G–I and Supplementary Figures 2B–D). Taken together, ZI PV neurons also negatively regulate scratching behaviors evoked by chronic itch.

## Discussion

Our study showed that ZI PV neurons displayed elevated activity related to acute itch-induced scratching. Chemogenetic activation of ZI PV neurons attenuated scratching behavior induced by both histamine and chloroquine. These phenomena were also seen under the condition of AD-induced chronic itch. These results together suggest that ZI PV neurons act as an endogenous negative modulatory center during itch stimuli.

Pain and itch are two opposing sensations. Pain attenuates itch sensation, and the suppression of pain could enhances itch (Ma, 2010; LaMotte et al., 2014). This was originally attributed to distinct neural circuit mechanisms at the spinal level (Davidson and Giesler, 2010). Then, subsequent evidence identified several supra-spinal structures related to this phenomenon. Spinal-projecting neurons in the S1 exert inhibitory effects on itch transmission *via* spinal inhibitory interneurons, but facilitate mechanical allodynia *via* spinal excitatory interneurons during neuropathic pain (Liu et al., 2018; Wu et al., 2021). Pain and itch are also subject to opposing descending modulation. The activation of PAG glutamatergic neurons suppresses nociception but facilitates itch (Samineni et al., 2017, 2019). This phenomenon may be owing to the dual modulation of downstream 5-HT signaling in itch and pain during the vicious itch-scratching cycle. Scratching could elicit pain, and consequently result in the activation of 5-HT neurons in the RVM. The released 5-HT acts upon two SDH neuronal subpopulations. Those expressing 5-HT_1*A*_R alone inhibit nociceptive processing, while the others expressing both gastrin releasing peptide receptor and 5-HT_1*A*_R facilitate itch transmission (Zhao et al., 2014). Herein, we identify a novel diencephalic area that affects itch and pain in opposing manners, and the activation of ZI PV neurons facilitates nocifensive behaviors (Wang et al., 2020a) but inhibits itch processing as seen in the present study. Distinct downstream neural pathways or postsynaptic neuronal types may mediate these opposing effects.

The unidirectional modulatory effect of ZI PV neurons adds the complexity of neurocircuitry mechanisms underlying itch processing. As shown in the present study, the activation of ZI PV neurons inhibits both histamine and chloroquine induced scratching, however, the inhibition of them fails to enhance scratching behavior. One possible explanation for this phenomenon is that as an exogenous, non-physiological manipulation way during itch stimuli, chemogenetic inhibition of these neurons may recruit other itch-suppressing pathways in a compensatory style, which masked the itch-facilitatory effect induced by decreased ZI PV neuronal activities themselves.

ZI provides massive projections toward various cortical and subcortical regions, including the S1, intra-laminar and higher-order thalamic nuclei, PAG, anterior pretectal nucleus, pontine reticular nucleus, and RVM (Mitrofanis, 2005; Zhao et al., 2019). Among them, the posterior thalamic nucleus (Po) represents a potential target of ZI for pain processing. Incertal neurons participate in a feed forward inhibitory circuit that blocking sensory transmission through Po, and neuropathic pain is associated with reduced ZI activity and consequently increased Po activity (Trageser and Keller, 2004; Masri et al., 2009). In addition, the activation of ZI-Po pathway mediates the analgesic effects of motor cortex stimulation (Cha et al., 2013). Emerging evidence suggest that Po mediates histaminergic itch (Zhu et al., 2020) and ZIv PV neurons send GABAergic projections to Po (Wang et al., 2020a). Thus, incertal projections toward Po might mediate the anti-pruritus effect of ZIv PV neurons. It is worth mentioning that PAG also receives inputs from ZIv PV neurons (Wang et al., 2020a). It is plausible that the descending PAG-RVM-SDH pathway may subserve the distinct roles of ZIv PV neurons in the modulation of itch and pain.

As a highly heterogenous nucleus, ZI possesses diverse neuronal types with capacity to release neurotransmitters including GABA, glutamate, and neuropeptides (Kolmac and Mitrofanis, 1999; Mitrofanis et al., 2004). ZI GABAergic neurons are abundant in number, which could be further divided by the expression of somatostatin, vasopressin, and PV (Watson et al., 2014). Distinct ZI sectors or neuronal types display diverse connectivity patterns and contribute to multiple roles of ZI in various physiological functions (Mitrofanis, 2005). Despite early studies indicating ZI as an integrative hub for global regulation of physiological behaviors, in-depth dissections of contributions of specific subpopulations were not emphasized until in recent researches with state-of-the-art tract tracing and activity manipulation techniques (Mitrofanis, 2005; Wang et al., 2020b). For instance, the activation of ZIr GABAergic neurons reduced defensive behavior *via* direct projections toward PAG (Chou et al., 2018), which was opposite to that of activating ZIv PV neurons devoid of projections to PAG (Wang et al., 2019). In the domain of pain research, early studies based on non-specific manipulations of ZI activity suggest the antinociceptive role of ZI (Masri et al., 2009; Petronilho et al., 2012; Moon et al., 2016; Moon and Park, 2017), which might be mediated by ZId/r subpopulations that receive glutamatergic inputs from midcingulate cortex (Hu et al., 2019). However, ZI PV neurons positively control nocifensive behavior *via* the incerta-thalamic circuit (Wang et al., 2020a). Herein, although the inhibitory role of ZI PV neurons during itch processing was identified, we did not know whether other subpopulations were engaged in itch behaviors. It would be exciting to explore whether and how (including circuit and molecular mechanisms) other subpopulations are involved in itch regulation in the future.

Chronic itch renders a challenging symptom for clinicians to manage. Although many novel treatments including immunomodulators and drugs targeting at the neural system are identified, frustrated voices concerning its efficacy never diminish, owing to the lack of evidence from large-scale controlled trials as well as adverse effects (e.g., somnolence and weight gain) (Yosipovitch et al., 2018). In the absence of ideal antipruritics, neuromodulation emerges as a promising alternative for refractory itch disorders. Emerging neural targets have been identified along pruritic transmission or modulation pathway, including the spinal cord (Hill and Paraiso, 2015) and the sensorimotor cortex (Knotkova et al., 2013; Nakagawa et al., 2016; Thibaut et al., 2019). There is an ever-growing body of efforts, indicating the importance of ZI stimulation in clinical settings. Deep brain stimulation of ZI has been demonstrated to improve akinesia and bradykinesia in patients with Parkinson’s disease (Voges et al., 2002), proximal tremor in patients with multiple sclerosis (Nandi et al., 2002), as well as obsessive symptoms in patients with obsessive compulsive disorder (Mallet et al., 2002). Interestingly, Lu et al. (2021) noticed changes of human perception of experimental heat pain in subthalamic DBS patients, supporting ZI as a potential target for pain modulation. Herein, we showed that ZI PV neurons negatively regulate scratching behaviors evoked by chronic itch. These data may lay preclinical basis for the application of ZI stimulation in the treatment of pharmaco-resistant itch.

This study is not without flaws. Firstly, it is unknown whether pain and itch activate the same population of ZI PV neurons. This issue could be addressed by *in vivo* miniscope, miniature two-photon imaging or multi-channel extracellular recordings (de Groot et al., 2020; Zhang et al., 2021), which helps determine whether individual ZI PV neuron responds to both pain and itch stimuli. In addition, the utilization of the targeted recombination in active populations system in Fos-CreER transgenic mice (Sanders et al., 2019) allows to determine whether selective manipulation of itching-responsive ZI neurons influences pain behaviors. Secondly, nocifensive responses induced by scratching may also activate PV neurons in the ZI. To selectively detect neuronal activity by itch stimuli, a neck collar should be used to avoid scratching-induced activation.

## Conclusion

In summary, our data revealed a novel role of ZI PV neurons in controlling itch signal processing. These knowledge would extend our understanding of central mechanisms underlying itch sensation and provide clues to intervention strategies for chronic itch.

## Data Availability Statement

The raw data supporting the conclusions of this article will be made available by the authors, without undue reservation.

## Ethics Statement

The animal study was reviewed and approved by the Institutional Animal Care and Use Committee of the Fourth Military Medical University.

## Author Contributions

JH and SW conceived and designed the experiments, conceived the study, analyzed the data, and revised the manuscript. JL, YB, and YL performed the experiments, analyzed the data, and wrote the manuscript. QZ, YWZ, DL, YYZ, and JL assisted with animal studies and histology. JL, JG, and GC performed and interpreted the data and statistical analyses. HT analyzed the data and revised the manuscript. All authors read and approved the final manuscript.

## Conflict of Interest

The authors declare that the research was conducted in the absence of any commercial or financial relationships that could be construed as a potential conflict of interest.

## Publisher’s Note

All claims expressed in this article are solely those of the authors and do not necessarily represent those of their affiliated organizations, or those of the publisher, the editors and the reviewers. Any product that may be evaluated in this article, or claim that may be made by its manufacturer, is not guaranteed or endorsed by the publisher.

## References

[B1] Cha M. Ji Y. Masri R. (2013). Motor cortex stimulation activates the incertothalamic pathway in an animal model of spinal cord injury. *J. Pain* 14 260–269. 10.1016/j.jpain.2012.11.007 23332495 PMC3594418

[B2] Chen Z. F. (2021). A neuropeptide code for itch. *Nat. Rev. Neurosci.* 22 758–776. 10.1038/s41583-021-00526-9 34663954 PMC9437842

[B3] Chou X. L. Wang X. Zhang Z. G. Shen L. Zingg B. Huang J. (2018). Inhibitory gain modulation of defense behaviors by zona incerta. *Nat. Commun.* 9:1151. 10.1038/s41467-018-03581-6 29559622 PMC5861117

[B4] Coggeshall R. E. (2005). Fos, nociception and the dorsal horn. *Prog. Neurobiol.* 77 299–352. 10.1016/j.pneurobio.2005.11.002 16356622

[B5] Davidson S. Giesler G. J. (2010). The multiple pathways for itch and their interactions with pain. *Trends Neurosci.* 33 550–558. 10.1016/j.tins.2010.09.002 21056479 PMC2991051

[B6] de Groot A. van den Boom B. J. van Genderen R. M. Coppens J. van Veldhuijzen J. Bos J. (2020). NINscope, a versatile miniscope for multi-region circuit investigations. *Elife* 9. 10.7554/eLife.49987 31934857 PMC6989121

[B7] Dong X. Dong X. (2018). Peripheral and Central Mechanisms of Itch. *Neuron* 98 482–494. 10.1016/j.neuron.2018.03.023 29723501 PMC6022762

[B8] Drzezga A. Darsow U. Treede R. D. Siebner H. Frisch M. Munz F. (2001). Central activation by histamine-induced itch: analogies to pain processing: a correlational analysis of O-15 H2O positron emission tomography studies. *Pain* 92 295–305. 10.1016/s0304-3959(01)00271-8 11323151

[B9] Gao Z. R. Chen W. Z. Liu M. Z. Chen X. J. Wan L. Zhang X. Y. (2019). Tac1-Expressing Neurons in the Periaqueductal Gray Facilitate the Itch-Scratching Cycle *via* Descending Regulation. *Neuron* 101 45.e–59.e. 10.1016/j.neuron.2018.11.010 30554781

[B10] Greaves M. W. Khalifa N. (2004). Itch: more than skin deep. *Int. Arch. Allergy Immunol.* 135 166–172. 10.1159/000080898 15375326

[B11] Hill A. J. Paraiso M. F. (2015). Resolution of Chronic Vulvar Pruritus With Replacement of a Neuromodulation Device. *J. Minim. Invasive Gynecol.* 22 889–891. 10.1016/j.jmig.2015.02.020 25757813

[B12] Hu T. T. Wang R. R. Du Y. Guo F. Wu Y. X. Wang Y. (2019). Activation of the Intrinsic Pain Inhibitory Circuit from the Midcingulate Cg2 to Zona Incerta Alleviates Neuropathic Pain. *J. Neurosci.* 39 9130–9144. 10.1523/jneurosci.1683-19.2019 31604834 PMC6855685

[B13] Kim B. S. Siracusa M. C. Saenz S. A. Noti M. Monticelli L. A. Sonnenberg G. F. (2013). TSLP elicits IL-33-independent innate lymphoid cell responses to promote skin inflammation. *Sci. Transl. Med.* 5:170ra116. 10.1126/scitranslmed.3005374 23363980 PMC3637661

[B14] Kim D. Kobayashi T. Nagao K. (2019). Research Techniques Made Simple: mouse Models of Atopic Dermatitis. *J. Invest. Dermatol.* 139 984.e–990.e. 10.1016/j.jid.2019.02.014 31010529 PMC6555635

[B15] Knotkova H. Portenoy R. K. Cruciani R. A. (2013). Transcranial direct current stimulation (tDCS) relieved itching in a patient with chronic neuropathic pain. *Clin. J. Pain* 29 621–622. 10.1097/AJP.0b013e31826b1329 23328331

[B16] Koch S. C. Acton D. Goulding M. (2018). Spinal Circuits for Touch, Pain, and Itch. *Annu. Rev. Physiol.* 80 189–217. 10.1146/annurev-physiol-022516-034303 28961064 PMC5891508

[B17] Kolmac C. Mitrofanis J. (1999). Distribution of various neurochemicals within the zona incerta: an immunocytochemical and histochemical study. *Anat. Embryol.* 199 265–280. 10.1007/s004290050227 10068092

[B18] LaMotte R. H. Dong X. Ringkamp M. (2014). Sensory neurons and circuits mediating itch. *Nat. Rev. Neurosci.* 15 19–31. 10.1038/nrn3641 24356071 PMC4096293

[B19] Li Y. Zhong W. Wang D. Feng Q. Liu Z. Zhou J. (2016). Serotonin neurons in the dorsal raphe nucleus encode reward signals. *Nat. Commun.* 7:10503. 10.1038/ncomms10503 26818705 PMC4738365

[B20] Li Z. Rizzi G. Tan K. R. (2021). Zona incerta subpopulations differentially encode and modulate anxiety. *Sci. Adv.* 7:eabf6709. 10.1126/sciadv.abf6709 34516764 PMC8442884

[B21] Liu K. Kim J. Kim D. W. Zhang Y. S. Bao H. Denaxa M. (2017). Lhx6-positive GABA-releasing neurons of the zona incerta promote sleep. *Nature* 548 582–587. 10.1038/nature23663 28847002 PMC5958617

[B22] Liu Y. Latremoliere A. Li X. Zhang Z. Chen M. Wang X. (2018). Touch and tactile neuropathic pain sensitivity are set by corticospinal projections. *Nature* 561 547–550. 10.1038/s41586-018-0515-2 30209395 PMC6163083

[B23] Lu C. W. Harper D. E. Askari A. Willsey M. S. Vu P. P. Schrepf A. D. (2021). Stimulation of zona incerta selectively modulates pain in humans. *Sci. Rep.* 11:8924. 10.1038/s41598-021-87873-w 33903611 PMC8076305

[B24] Ma Q. (2010). Labeled lines meet and talk: population coding of somatic sensations. *J. Clin. Invest.* 120 3773–3778. 10.1172/jci43426 21041959 PMC2964985

[B25] Mallet L. Mesnage V. Houeto J. L. Pelissolo A. Yelnik J. Behar C. (2002). Compulsions, Parkinson’s disease, and stimulation. *Lancet* 360 1302–1304. 10.1016/s0140-6736(02)11339-0 12414208

[B26] Masri R. Quiton R. L. Lucas J. M. Murray P. D. Thompson S. M. Keller A. (2009). Zona incerta: a role in central pain. *J. Neurophysiol.* 102 181–191. 10.1152/jn.00152.2009 19403748 PMC2712264

[B27] Mitrofanis J. (2005). Some certainty for the “zone of uncertainty”? Exploring the function of the zona incerta. *Neuroscience* 130 1–15. 10.1016/j.neuroscience.2004.08.017 15561420

[B28] Mitrofanis J. Ashkan K. Wallace B. A. Benabid A. L. (2004). Chemoarchitectonic heterogeneities in the primate zona incerta: clinical and functional implications. *J. Neurocytol.* 33 429–440. 10.1023/B:NEUR.0000046573.28081.dd 15520528

[B29] Moon H. C. Lee Y. J. Cho C. B. Park Y. S. (2016). Suppressed GABAergic signaling in the zona incerta causes neuropathic pain in a thoracic hemisection spinal cord injury rat model. *Neurosci. Lett.* 632 55–61. 10.1016/j.neulet.2016.08.035 27561604

[B30] Moon H. C. Park Y. S. (2017). Reduced GABAergic neuronal activity in zona incerta causes neuropathic pain in a rat sciatic nerve chronic constriction injury model. *J. Pain Res.* 10 1125–1134. 10.2147/jpr.s131104 28546770 PMC5436785

[B31] Nakagawa K. Mochizuki H. Koyama S. Tanaka S. Sadato N. Kakigi R. (2016). A transcranial direct current stimulation over the sensorimotor cortex modulates the itch sensation induced by histamine. *Clin. Neurophysiol.* 127 827–832. 10.1016/j.clinph.2015.07.003 26190177

[B32] Nandi D. Chir M. Liu X. Bain P. Parkin S. Joint C. (2002). Electrophysiological confirmation of the zona incerta as a target for surgical treatment of disabling involuntary arm movements in multiple sclerosis: use of local field potentials. *J. Clin. Neurosci.* 9 64–68. 10.1054/jocn.2001.1012 11749021

[B33] Papoiu A. D. Coghill R. C. Kraft R. A. Wang H. Yosipovitch G. (2012). A tale of two itches. Common features and notable differences in brain activation evoked by cowhage and histamine induced itch. *Neuroimage* 59 3611–3623. 10.1016/j.neuroimage.2011.10.099 22100770 PMC3288667

[B34] Peters K. Z. Cheer J. F. Tonini R. (2021). Modulating the Neuromodulators: dopamine, Serotonin, and the Endocannabinoid System. *Trends Neurosci.* 44 464–477. 10.1016/j.tins.2021.02.001 33674134 PMC8159866

[B35] Petronilho A. Reis G. M. Dias Q. M. Fais R. S. Prado W. A. (2012). Antinociceptive effect of stimulating the zona incerta with glutamate in rats. *Pharmacol. Biochem. Behav.* 101 360–368. 10.1016/j.pbb.2012.01.022 22327011

[B36] Samineni V. K. Grajales-Reyes J. G. Copits B. A. O’Brien D. E. Trigg S. L. Gomez A. M. (2017). Divergent Modulation of Nociception by Glutamatergic and GABAergic Neuronal Subpopulations in the Periaqueductal Gray. *eNeuro* 4:ENEURO.0129-16.2017. 10.1523/eneuro.0129-16.2017 28374016 PMC5370278

[B37] Samineni V. K. Grajales-Reyes J. G. Grajales-Reyes G. E. Tycksen E. Copits B. A. Pedersen C. (2021). Cellular, circuit and transcriptional framework for modulation of itch in the central amygdala. *Elife* 10:e68130. 10.7554/eLife.68130 34032210 PMC8172243

[B38] Samineni V. K. Grajales-Reyes J. G. Sundaram S. S. Yoo J. J. Gereau R. W. T. (2019). Cell type-specific modulation of sensory and affective components of itch in the periaqueductal gray. *Nat. Commun.* 10:4356. 10.1038/s41467-019-12316-0 31554789 PMC6761157

[B39] Sanders K. M. Sakai K. Henry T. D. Hashimoto T. Akiyama T. (2019). A Subpopulation of Amygdala Neurons Mediates the Affective Component of Itch. *J. Neurosci.* 39 3345–3356. 10.1523/jneurosci.2759-18.2019 30819800 PMC6788830

[B40] Schweinhardt P. Bushnell M. C. (2010). Pain imaging in health and disease–how far have we come? *J. Clin. Invest.* 120 3788–3797. 10.1172/jci43498 21041961 PMC2964988

[B41] Su X. Y. Chen M. Yuan Y. Li Y. Guo S. S. Luo H. Q. (2019). Central Processing of Itch in the Midbrain Reward Center. *Neuron* 102 858.e–872.e. 10.1016/j.neuron.2019.03.030 31000426

[B42] Tan L. L. Kuner R. (2021). Neocortical circuits in pain and pain relief. *Nat. Rev. Neurosci.* 22 458–471. 10.1038/s41583-021-00468-2 34127843

[B43] Tey H. L. Yosipovitch G. (2011). Targeted treatment of pruritus: a look into the future. *Br. J. Dermatol.* 165 5–17. 10.1111/j.1365-2133.2011.10217.x 21219293 PMC3125418

[B44] Thibaut A. Ohrtman E. A. Morales-Quezada L. Simko L. C. Ryan C. M. Zafonte R. (2019). Distinct behavioral response of primary motor cortex stimulation in itch and pain after burn injury. *Neurosci. Lett.* 690 89–94. 10.1016/j.neulet.2018.10.013 30312754 PMC8279808

[B45] Trageser J. C. Keller A. (2004). Reducing the uncertainty: gating of peripheral inputs by zona incerta. *J. Neurosci.* 24 8911–8915. 10.1523/jneurosci.3218-04.2004 15470158 PMC1388274

[B46] Treede R. D. Kenshalo D. R. Gracely R. H. Jones A. K. (1999). The cortical representation of pain. *Pain* 79 105–111. 10.1016/s0304-3959(98)00184-5 10068155

[B47] Venkataraman A. Brody N. Reddi P. Guo J. Gordon Rainnie D. Dias B. G. (2019). Modulation of fear generalization by the zona incerta. *Proc. Natl. Acad. Sci. U.S.A.* 116 9072–9077. 10.1073/pnas.1820541116 30967506 PMC6500173

[B48] Voges J. Volkmann J. Allert N. Lehrke R. Koulousakis A. Freund H. J. (2002). Bilateral high-frequency stimulation in the subthalamic nucleus for the treatment of Parkinson disease: correlation of therapeutic effect with anatomical electrode position. *J. Neurosurg.* 96 269–279. 10.3171/jns.2002.96.2.0269 11838801

[B49] Wang X. Chou X. L. Zhang L. I. Tao H. W. (2020b). Zona Incerta: an Integrative Node for Global Behavioral Modulation. *Trends Neurosci.* 43 82–87. 10.1016/j.tins.2019.11.007 31864676 PMC7439563

[B50] Wang H. Dong P. He C. Feng X. Y. Huang Y. Yang W. W. (2020a). Incerta-thalamic Circuit Controls Nocifensive Behavior *via* Cannabinoid Type 1 Receptors. *Neuron* 107 538.e–551.e. 10.1016/j.neuron.2020.04.027 32502461

[B51] Wang X. Chou X. Peng B. Shen L. Huang J. J. Zhang L. I. (2019). A cross-modality enhancement of defensive flight *via* parvalbumin neurons in zona incerta. *Elife* 8:e42728. 10.7554/eLife.42728 30985276 PMC6486150

[B52] Watson C. Lind C. R. Thomas M. G. (2014). The anatomy of the caudal zona incerta in rodents and primates. *J. Anat.* 224 95–107. 10.1111/joa.12132 24138151 PMC3969054

[B53] Weisshaar E. Matterne U. (2016). Epidemiology of itch. In: itch: mechanisms and Treatment. *Curr. Probl. Dermatol.* 50 11–17. 27578064 10.1159/000446010

[B54] Wu Z. H. Shao H. Y. Fu Y. Y. Wu X. B. Cao D. L. Yan S. X. (2021). Descending Modulation of Spinal Itch Transmission by Primary Somatosensory Cortex. *Neurosci. Bull.* 37 1345–1350. 10.1007/s12264-021-00713-9 34057697 PMC8423972

[B55] Yosipovitch G. Rosen J. D. Hashimoto T. (2018). Itch: from mechanism to (novel) therapeutic approaches. *J. Allergy Clin. Immunol.* 142 1375–1390. 10.1016/j.jaci.2018.09.005 30409247

[B56] Yuan L. Liang T. Y. Deng J. Sun Y. G. (2018). Dynamics and Functional Role of Dopaminergic Neurons in the Ventral Tegmental Area during Itch Processing. *J. Neurosci.* 38 9856–9869. 10.1523/jneurosci.1483-18.2018 30266741 PMC6596242

[B57] Zhang C. Zhu H. Ni Z. Xin Q. Zhou T. Wu R. (2021). Dynamics of a disinhibitory prefrontal microcircuit in controlling social competition. *Neuron.* 10.1016/j.neuron.2021.10.034 34793692

[B58] Zhao Z. D. Chen Z. Xiang X. Hu M. Xie H. Jia X. (2019). Zona incerta GABAergic neurons integrate prey-related sensory signals and induce an appetitive drive to promote hunting. *Nat. Neurosci.* 22 921–932. 10.1038/s41593-019-0404-5 31127258

[B59] Zhao Z. Q. Liu X. Y. Jeffry J. Karunarathne W. K. Li J. L. Munanairi A. (2014). Descending control of itch transmission by the serotonergic system *via* 5-HT1A-facilitated GRP-GRPR signaling. *Neuron* 84 821–834. 10.1016/j.neuron.2014.10.003 25453842 PMC4254557

[B60] Zhou M. Liu Z. Melin M. D. Ng Y. H. Xu W. Südhof T. C. (2018). A central amygdala to zona incerta projection is required for acquisition and remote recall of conditioned fear memory. *Nat. Neurosci.* 21 1515–1519. 10.1038/s41593-018-0248-4 30349111 PMC7261007

[B61] Zhu Y. B. Xu L. Wang Y. Zhang R. Wang Y. C. Li J. B. (2020). Posterior Thalamic Nucleus Mediates Facial Histaminergic Itch. *Neuroscience* 444 54–63. 10.1016/j.neuroscience.2020.07.048 32750381

