# Supplementary material for: Parvalbumin Neurons in Zona Incerta Regulate Itch in Mice
Source: Front Mol Neurosci. 2022 Mar 1;15:843754. doi: 10.3389/fnmol.2022.843754 (PMC8920991; doi:10.3389/fnmol.2022.843754)
Supplement: Supplementary file 1 [file Image_1.pdf]

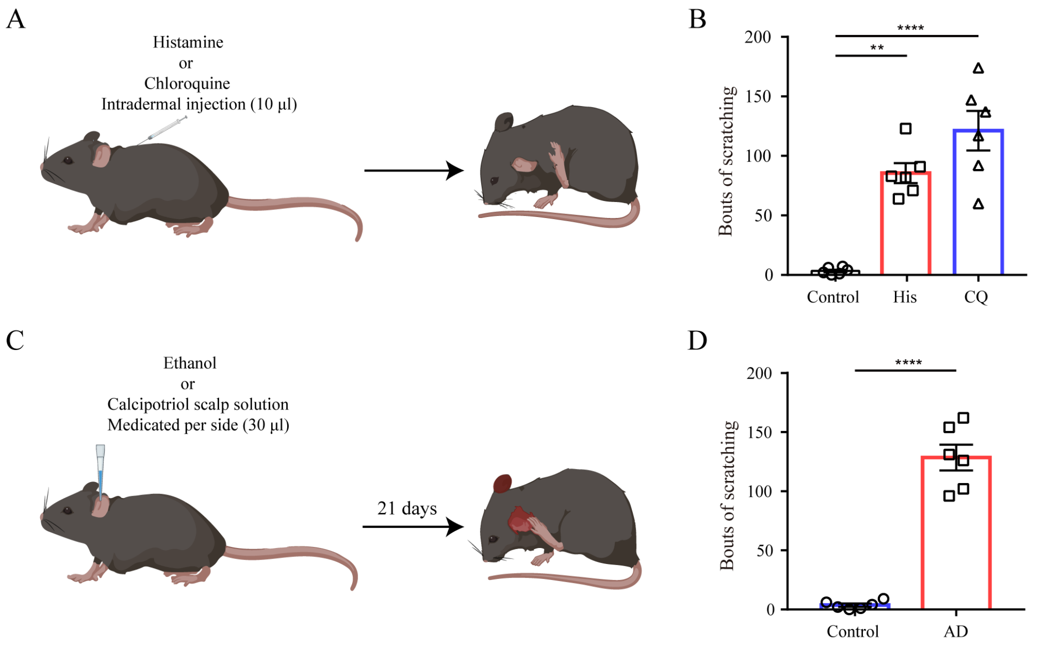

Supplemental Figure 1: (A) Schematic of the establishment of histamine and chloroquine induced acute itch models. (B) Intradermal injections of histamine and chloroquine induce robust scratching behavior in mice. Kruskal-Wallis test and Nemenyi multiple comparisons tests. $\chi 2=12.538$, $P=0.002$. n=6 mice in each group. (C) Schematic of the establishment of atopic dermatitis (AD) induced chronic itch model. (D) Consecutive tropical application of calcipotriol induces robust scratching behavior in mice. Unpaired T test. $t=-11.394$, $P<0.001$. n=6 mice in each group. ****$P<0.0001$,***$P<0.001$, **$P<0.01$.

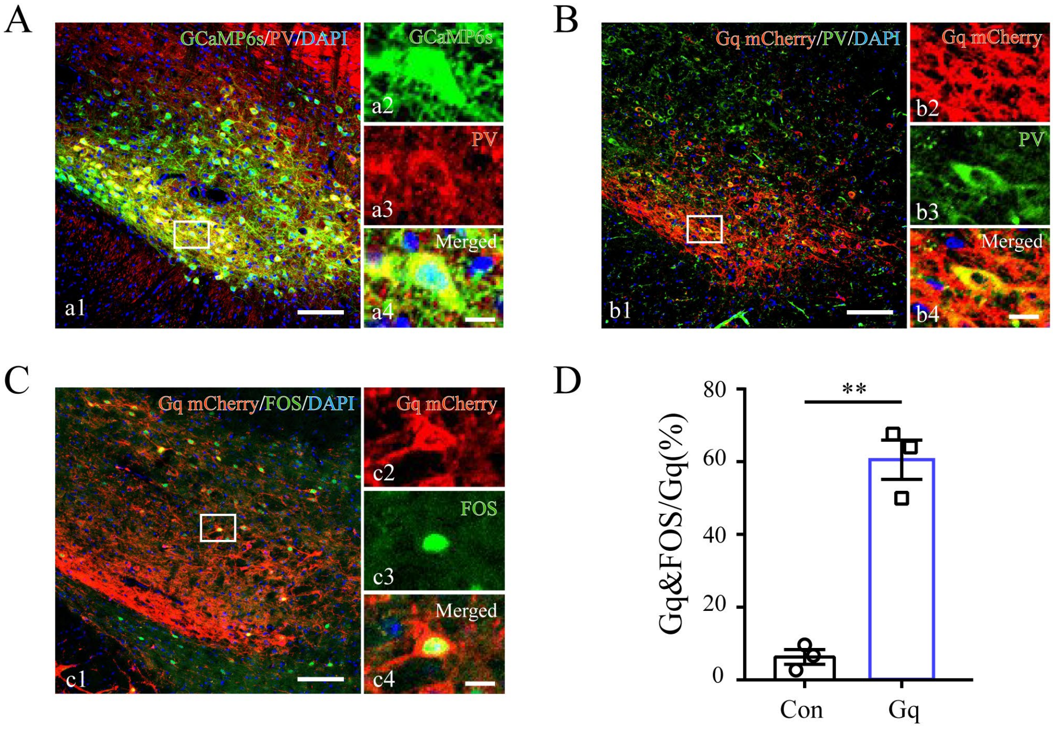

Supplemental Figure 2: (A) Representative photographs showing the expression of PV (red) in GCaMP6s-positive (green) neurons in the ZI of PV-Cre mice with atopic dermatitis. (B) Representative photographs showing the expression of PV (green) in GCaMP6s-positive (red) neurons in the ZI of PV-Cre mice with atopic dermatitis. (C) Representative photographs showing the expression of FOS (green) in hM3Dq-positive (red) neurons in the ZI of PV-Cre mice with atopic dermatitis. (D) Quantification of FOS-expressing neurons in mice with AAV-hM3Dq and AAV-mCherry injection showed CNO injection increased FOS expression in ZI PV neurons in atopic dermatitis mice of AAV-hM3Dq group. n = 3 mice per group, 3 sections per mouse. Unpaired T test. t=-9.433, P=0.001. The framed area in a1, b1, and c1 was magnified in a2-4, b2-4, and c2-4, respectively. Scale bars represent 100 μm in a1, b1, and c1, and 50 μm in a2-4, b2-4, and c2-4. **P<0.01.
